# The Benefits of Improving Animal Welfare from the Perspective of Livestock Stakeholders across Asia

**DOI:** 10.3390/ani9040123

**Published:** 2019-03-28

**Authors:** Michelle Sinclair, Claire Fryer, Clive J. C. Phillips

**Affiliations:** Centre for Animal Welfare and Ethics, School of Veterinary Sciences, The University of Queensland, Gatton, QLD 4343, Australia; c.fryer1@uq.edu.au (C.F.); c.phillips@uq.edu.au (C.J.C.P.)

**Keywords:** animal welfare, benefit, profit, human health, Asia, livestock

## Abstract

**Simple Summary:**

A previous study into successful international animal welfare management strategy presented the vital need for animal welfare proponents to establish mutual benefits with the livestock industry. What the perceived benefits to addressing farm animal welfare are, is therefore important information not previously researched. This study asked leaders in the livestock industry in regions across six Asian countries what they saw as the key benefits for improving animal welfare, and which of those benefits they found the most compelling. The potentials to increase productivity of the animals and improve meat quality were among the most frequently cited and most highly rated across the countries. Important differences in the focus of other benefits existed by country, with food safety of highest importance in China and Vietnam, and people-focused benefits (such as human health and improved community livelihood) of greater importance in countries with higher rates of poverty such as India and Bangladesh. Animal-based reasons, such as improving animal welfare to the benefit of the animals themselves, were not compelling benefits in any of the investigated countries, other than India. The results of this study could assist in the development of improved animal welfare strategies.

**Abstract:**

In this study, 17 focus group meetings were held with livestock industry leaders in geographically dispersed areas of China, Vietnam, Thailand, Malaysia, India and Bangladesh, regarding animal welfare issues, potential solutions and attitudes. Livestock leaders were asked ‘what do you see as the benefits to improving animal welfare’ and later to discuss the potential benefits and rank them according to their associated importance. While differences existed by country, the most important perceived benefit area across all countries was financial in nature, primarily focussed on the potential to increase the productive output of the animals and to improve meat and product quality. However, doubt existed around the ability to increase profit against the cost of improving animal welfare, particularly in China. Human health benefits and the tie to human welfare and community livelihood were considered most important in India and Bangladesh, and animal-focussed benefits were not significant in any countries, except India and, to a lesser extent, Bangladesh. Thus, improving animal welfare for the sake of the animals is unlikely to be a compelling argument. The results presented here can be used to create meaningful mutual ground between those that advocate improvement of animal welfare and the stakeholders that have the ability to implement it, i.e., the livestock industry.

## 1. Introduction 

Farm animal production is arguably the most economically important interface between humans and other animals on this planet. It has the potential to cause suffering in large numbers of animals, over prolonged periods of time, ending in a death that has the conceivable ability to epitomise that suffering. As an industry that has systematically experienced rapid growth and intensification in most regions of the world, methods employed during farming, transport and slaughter are frequently the focus of public concern and advocacy lobby. Ethical arguments exist for addressing farm animal welfare, including it being ‘the right thing to do’ for the animals themselves; however, these arguments may not be compelling to all important parties engaged in this sector. Depending on the area of the world, other influential factors make more compelling motivators [1], and the literature on this point paints a much more complex landscape than one of basic ethical value, particularly in emerging countries [2,3]. Animal agriculture is not simply a theoretical interface between humans and other species, it is an economic endeavour; it functions foremost as a business, and the stakeholders in the position to have the most power over the welfare of the animals in the sector are those working within the livestock industry. 

Key tenets of successful international animal welfare initiatives have been outlined in a recent study, which emphasized the importance of engaging with the industry and establishing mutual benefits as a basis for collaboration [4]. According to the literature, apart from the obvious benefits to the animals themselves, the espoused benefits for improving animal welfare vary. One study focuses on the fact that economic benefits have been historically omitted from consideration and that economists, amongst others, should play an important role when developing animal welfare initiatives [5].

From an economic perspective, improving product quality and reducing animal losses are the potential benefits to improving animal welfare that are found in the scientific animal welfare literature most frequently. Mitigating losses through reduced mortality [6], reducing damage to carcasses through reducing bruising, injuries and the incidence of pale soft meat (PSE) in pigs [7] and dark cutting (dark firm and dry) [8] in beef cattle—both signs of significant stresses caused to the animal before its death—are cited as key benefits of addressing animal welfare concerns [9]. Some studies also cite improved productivity of animals [10], as well as improved reproduction and thrift in livestock [11]. Apart from the product-based economic benefits, there are the strategic business benefits. One benefit that does not appear to have been contested is that improving welfare offers commercial opportunities to market products as being from higher welfare systems, with some studies showing that consumers are willing to pay more (however, not vast amounts more) to purchase meat that makes them feel better about the life the animal had [12]. This, however, is based on studies mostly conducted in western nations, and this may vary in less developed countries where consumers need to buy food as cheaply as possible and do not have the luxury of being discerning. Having noted that, this could still remain a relevant benefit for enterprises in developing countries that are seeking to export, or continue exporting, animal products to western nations. 

A major benefit identified in the scientific literature centers on the notion that the public, as evident in many parts of the world, is demanding better treatment of animals [13]. Improving animal welfare offers the business benefit of mitigation of risk to the brand through bad publicity, loss of purchase partnerships and even the jeopardy of a whole industry. In some parts of the world, this is a concern, fuelled by advocacy lobby efforts that have seen reformation of farming practices, such as the abolition of veal crates, cage eggs and sow stalls in the European Union [14], and it has likewise caused periodic market collapses, such as that experienced by the live export industry in Australia [15]. In addition to avoiding poor publicity, improving animal welfare also provides positive marketing, which has the opportunity to improve the public’s perception of the livestock industries as a whole [13]. Finally, in terms of business benefits, the scientific literature identifies employment benefits. By going through 10 years of industry data, it was discovered that improved animal welfare makes the animals safer and easier to handle, which results in a need for fewer staff, who are more satisfied, likely to have substantially less time off and have less medical expenses [9,16].

Apart from the benefits received by the animals directly from improving their animal welfare and the business and economic benefits, some wider community-based social benefits are also reported in the scientific literature [17,18]. This includes mitigating environmental despoliation and mitigating the non-therapeutic use of antibiotics driving the emerging anti-microbial resistance crisis. A close connection between animal and human welfare has been advanced under the umbrella of the One Welfare concept [19]. As a demonstration of that, attention has been drawn to the risks to human health of operating in environments that are poor for animal welfare, including the incubation of pathogens found in high-confinement situations, along with respiratory problems and low-level antibiotic resistance [20]. One study has even found that where animal welfare has become a priority, it has contributed to positive competition within communities to improve the health and strength of the animals they care for [21].

Despite the potential benefits of improving the animal welfare of farmed animals, introducing these changes can be expensive, and some of the scientific literature is cautious of overstating the economic benefits awarded [9]. This is particularly true when it comes to space allowances and stocking density, where profits may be increased by maintaining more animals to a smaller space, however detrimental this is to the welfare of the individual animal and even if it requires animals to be pushed beyond their biological limits through selective breeding or husbandry practices such as introducing medication and chemical supplementation [9]. 

It has been argued that, while animal welfare improvements are often perceived to conflict with economic gain, which causes hesitation within the industry, modelling financial benefits may provide compelling motivation to overcome that perception [6]. Considering the costs of implementing higher welfare systems, cost benefit analyses have found that the total income potential was still increased [12,22,23]. 

Because most of the literature is western-based, it is not clear whether the livestock stakeholders’ perception of benefits vary in other parts of the world, in particular in developing countries. Asia is home to the biggest livestock-producing country in the world, the People’s Republic of China (henceforth referred to as China) [24], and no animal welfare legislation exists. Globalisation of society requires that we assess stakeholder perceptions and understand their priorities in major livestock-producing nations in order to provide incentives that make solutions realistically attainable [5]. For those involved in governance, domestic enterprise, export/import business enterprise, or animal welfare advocacy, understanding the potential benefits of addressing animal welfare, as perceived (or not perceived) by livestock stakeholders, provides an important step in identifying mutual benefits to create partnerships, improve initiatives and/or enact policy reform across borders. This study begins addressing this gap by reporting the outcomes of a series of focus groups held across six culturally diverse countries in Asia that addressed the issue of what benefits might derive from improvements in animal welfare. 

## 2. Methods and Materials

To gather data for this project, 17 focus groups were held in geographically dispersed locations across Vietnam, Malaysia, Thailand, China, India and Bangladesh (see Table 1). Locations were chosen in different areas of each country (i.e., south, north, central, capital and regional) in an effort to capture potential varied sentiments between domestic regions. Industry leaders were invited as representatives for the livestock industry to discuss the state of animal welfare in their country, in the context of major welfare issues, challenges, solutions, opportunities and perceived benefits to improving animal welfare, with the benefits being the focus of this paper. 

Participants were invited through country-based collaborators based on the following selection criteria: that they were leaders in the animal production sector, working for an organisation with a maximum of two government vets and with the ability to implement change into private businesses (see Table 2). The majority were private industry leaders (e.g., pig or poultry slaughterhouse, or production managers or owners). In some groups, some participants were known as professional colleagues. Although plans were made for 5 to 7 participants in each session, the actual number of participants present on the day varied from 3 to 14, with some participants cancelling and others indicating their desire to attend just before the event.

In this instance, focus groups were selected as the method of data collection in preference of surveys due to their scope to collect broader qualitative data, which, once identified, can then be measured quantitatively in future studies. Likewise, focus groups were chosen in preference of individual interviews to enable the collection of data from a wider sample size, to encourage cross-participant discussion that may lead to more in-depth and honest data collection and to allow for frequent consensus checks where the facilitator can ascertain if sentiments are shared, or contested, by the participant group. Within the focus groups, participants were asked an open-ended question at the onset of the focus group by the lead researcher (MS), i.e., ‘what do you see as the benefits to addressing animal welfare’. Their collective round table responses were recorded and presented back to the participants later during an activity that invited a group discussion on ranking the benefits from most important to least important, as a group. Participants that felt strongly towards certain benefits chose to advocate for a higher ranking of those benefits, in discussion form, and the groups ultimately voted democratically by raising their hands to vote for the ‘most important benefit’ from top to bottom. The discussions surrounding the benefits of improving animal welfare and the final rankings delivered through the activity were documented and form the basis of this study.

The remainder of focus group content considered specific animal welfare issues, solutions, meanings and motivations, which will be presented in separate manuscripts. 

All contributions were voice-recorded during the sessions, and additional field notes were taken by a research assistant (CF). Both data sets were used to create abridged transcripts of each session. As participation was subject to translation by a third-party translator and then presented in English to the researchers, word for word transcripts were not possible.

The average time of sessions was 3.5 h, with an average of eight participants. Sessions with higher numbers of participants often ran approximately 30–45 min past the scheduled 3.5 h to offer all participants adequate opportunity to contribute. Transcripts were uploaded into NVivo software for Mac 11.4.3 for analysis.

### Analysis

Benefits were identified and coded as a primary node/theme in Nvivo. The benefits were then classified into broader categories depending on who or what they fundamentally benefitted (human benefit, business benefit, animal benefit or community benefits). Data were then divided into relevant logical sub-themes, where present, identified by careful inspection and familiarization of the data, and within each benefit, key quotes that demonstrate the sentiments towards that benefit were manually selected for inclusion in this paper. At the completion of analysis and coding of themes and sub-themes, no new benefits emerged from the data, suggesting data saturation. The same lead researcher (MS) that conducted the focus groups also coded all themes/nodes and conducted the analysis.

To avoid presenting misleading data, linguistics and tone are not reported, as all data were translated, abbreviated, and summarised through six translators, from six different languages to English. For this reason, rather than focussing on word usage, more attention was paid to the careful analysis on the key themes (benefits), the frequency of their appearance across countries, the general context and meanings that were applied to them by the participants and how they relate to one another. However, word frequency functions in NVivo were utilised in the identification of sub themes and were reported infrequently in the results. Direct quotes are presented in the results according to the country in which they were collected, with the abbreviated codes presented in Table 1.

This study was granted human ethics approval by the University of Queensland Ethics Committee, approval number: 2017000628.

## 3. Results

In general, stakeholder leaders were positive about animal welfare and forthcoming with potential benefits; however, it is important to note that, in some regions, the existence of the reported benefits was met with scepticism. In some groups (listed individually below), while benefits were raised as worthwhile, some participants were dubious about the ability to obtain the benefits by addressing animal welfare. This was particularly the case for benefits tied to economics and productivity. In some sessions, participants struggled to identify benefits to addressing animal welfare at all. On one rare occasion, addressing animal welfare was openly associated with liability and cost, rather than benefit. 

In Zhengzhou (CH), participants were not confident in listing any benefits, with one participant stating that ‘why some people don’t improve welfare is because of the limitation of economic (factors), not because of their consciousness’. 

It is impossible in this experimental setting to quantify how many individuals had this sentiment of doubt, as constructs such as conformity and groupthink play a role in the data that are shared; however, this sentiment could be valuably followed up with an individual-level study. For that reason, the following report represents general sentiments of scepticism only and is not a reflection of opinions shared across the country (or even across the entire group in some instances). 

In Chiang Mai (TL), one participant raised the point that they are doing well with animal welfare in their business; however, in the end, the price they receive for their product is the same as those who have practices that are bad for welfare. ‘It’s not very fair because when you do (improve) animal welfare you have to put in more effort, but you gain back just about the same’ <TL>. While some agreed with this comment, another participant stated that ‘if we take care of the animals well, you don’t need to spend much on the medical expense and so on, and then the costs will be reduced’ <TL>. Similar sentiments were also expressed regarding the existence of financial benefit in Guangzhou (China), with one participant stating ‘we want to know if there’s any specific data to prove a positive connection between animal welfare and economic benefits to company…if we have such data (it) will become much easier to promote (the) concept’ <CH>. On the other hand, in response, it was also stated that ‘if we don’t have an improvement in production rate or, even worse, production potential but we do have better flavour or meat quality, that is also acceptable’ <CH>.

These sentiments were also present in Negeri Sembilian (Malaysia), where a ‘conflict between making money and (animal) welfare’ was expressed. ‘They know if they take good care it will benefit them, but in terms of making profit with limited space and budget, the issue is that it is hard to make a profit’ <MAL>. ‘It needs a lot of investment not only in new facilities but also to improve old facilities to use better technology and housing. For us businessmen to improve animal welfare, or make any changes, we need money…at the same time when we improve animal welfare, we want to improve the output. Businessmen will rarely see the benefit’ <MAL>. By way of further example, one farm manager stated that ‘(if) the handling of chicken during harvest is more gentle, we can expect reduced processing time’, then added ‘damage (can) be reduced, that’s the benefit, but stakeholders might not understand this clearly yet…benefits are not clearly understood’ <MAL>.

Lastly, it was also acknowledged by one participant in Malaysia that the benefits that had been raised by the group along with their ranking would be ‘seen differently by NGOs, that it would be upside down’, and then commented (with agreement from the group) that NGOs don’t seem to understand the livestock industry, due to different goals and priorities. ‘The industry wants to make a profit, but the NGOs don’t, this issue will be questioned again and again’ <MAL>.

## 4. Nature of Benefit

In terms of species context, leaders in China and Vietnam mostly gave examples relating to poultry and pig production, in Malaysia and Thailand, the focus was primarily on poultry production, in Bangladesh, on cattle, goat and sheep, and in India, comments were less species-specific, with only rare comments regarding cattle (likely due to the beliefs of the Hindu population) and pigs (likely due to the beliefs of the Muslim community responsible for slaughter in India).

**Figure 1:**
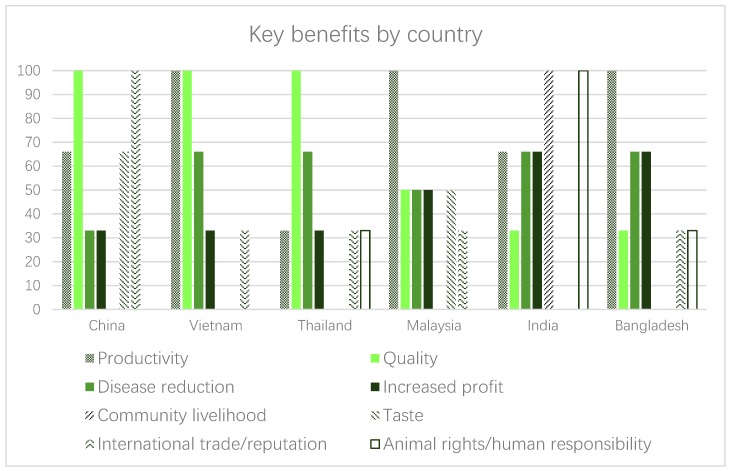
Comparison within countries regarding the appearance of certain perceived benefits for addressing animal welfare. Note: the values represent the % of focus groups that indicated the selected benefit within that country.

**Figure 2:**
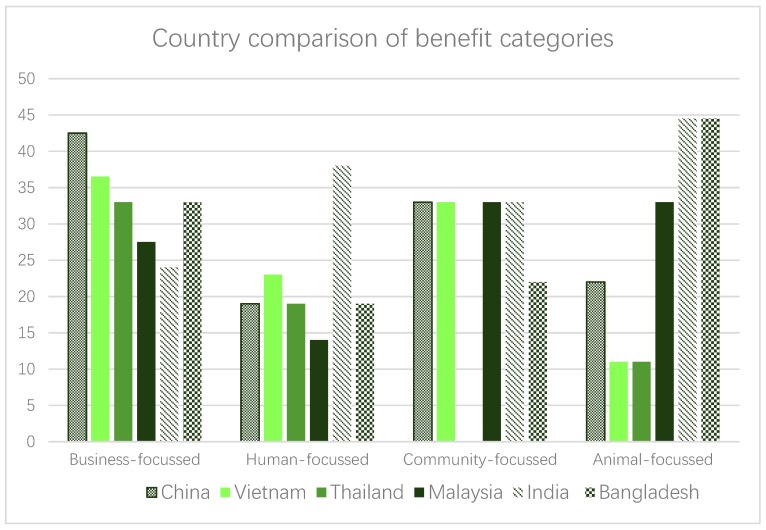
Comparison between countries of benefit categories, based on frequency of appearance. Note: Amount of times a benefit falling under this category appeared, presented in a percentage according to the amount of opportunities to raise it as a benefit per country. Individual benefits associated to categories as per the colour key in Table 1.

Table 3 quantifies and outlines all benefits identified by the livestock leaders, according to country and region, and the subsections below aim to provide further contextual information by presenting illustrative quotes around these benefits. Table 4 presents the outcome of an activity within the focus groups where leaders were asked to rank the benefits they presented at the onset of the session, in order of importance. Figure 1 presents the most frequently identified benefits by respondents in each country, while Figure 2 presents benefit categories, and providing country comparisons. Within Figure 2, all benefits were placed into categories based on their intended beneficiary (i.e., business, human, community, animal). Where the suitable category for a benefit was not clear, confirmation was sought from the original data.

### 4.1. Financial Benefits: Improved Animal Productivity

‘For businessmen, most of us we see that to improve animal welfare, or any changes, we need money’ <MAL>. ‘I think man is the most selfish creature, so revenue profit is number 1’ <IN>.

Although some aforementioned doubt existed as to the actual financial benefits (particularly when considering increased profit or return from investment in higher welfare systems), leaders were, however, mostly positive towards the existence of potential financial benefits. This was particularly the case with reducing economic losses (reduction of treatments and antibiotic usage) and with animal-based profit measures such as increasing the productivity of the animals themselves and improving the quality of the meat/animal product.

Improved animal productivity along with improved meat quality were the most important benefits identified. In regard to productivity, leaders made statements such as ‘when body condition is good, production is also high, so profit and productivity is increased <BA>’. ‘(I am) a farmer, (I) produce chicken and laying hens and observe (that) if chickens are given a good climate, good environment, ventilation, and space it improves (their) productivity’ <VN>. ‘(The birds) need to perform optimally in terms of productivity, that is why we make sure they are not too hot…happy birds make more money, that’s what we understand about welfare’ <MAL>. ‘When the pigs are very depressed or under stress they will grow slowly’ <CH>. After describing the situation of most stockholders in Bangladesh, in that animals frequently share houses with families, one participant stated (with general agreement amongst the group), that where the animals are given love and affection, when they come when their name is called and when psychological welfare is high ‘in that environment, the meat production is very high…the reproduction and meat production is great…it is the most important thing’ <BA>. 

### 4.2. Financial Benefits: Improved Meat and Product Quality

Along with improved productivity, meat quality was the most important benefit described. ‘I am a farmer of pork…feed, housing, water quality and slaughter…all these things improve meat quality, economic efficiency and value’ <VN>. ‘All species can be eaten, and most people treat them inhumanely especially in slaughterhouse, and to improve animal welfare can improve meat quality’ <VN>. ‘At slaughterhouse, (I) observed and realise if you improve handling, with stunning, it improves meat quality’ <VN>.

In Thailand; ‘I come from a slaughterhouse and I think that if they have good animal welfare, it reduces defects, the animals are more convenient and easier to handle, and it’s a good product’ <TL>. ‘The benefit for animal welfare is you will get a good quality product, reduced PSE, and also if you give (the animal) good welfare you will get a good yield’ <TL>. ‘To improve animal welfare means we will get good quality of products and customer will be happy with that’ <TL>. In China; ‘with the development of ecological agriculture, the importance of animal welfare has been emerging, so we need to improve both the management and give (the) animals some humane treatment to improve the quality of the products; that way we make the whole chain happy’ <CH>. ‘In (the) slaughterhouse, the brokers who buy and transports pigs have realised they need to rest the pigs for some days before slaughter, and the meat quality will become much better and taste good’ <CH>. ‘If we can have better animal welfare for the chicken, we can also have better benefits for our economy and our livelihood <CH>’.

### 4.3. Financial Benefits: Risk Avoidance And Business Loss Mitigation and Opportunity

In addition to the animal and product-based financial benefits, leaders also shared financial benefits in the form of risk and loss mitigation, through reducing the costs of medicine and treatments and lowering mortality. ‘Animal welfare requirements in standards should be satisfied from farm to slaughterhouse, even in the lairage and on the truck, feed, water, handling, all steps… (this will result in the) best quality and also improvement in health, it will reduce economic loss’ <VN>. ‘Improving animal welfare will improve animal health, less disease, lower mortality and improve growth rate (to) improve economic efficiency’ <VN>.

The business risk for the domestic brand and product sales of not addressing animal welfare was also raised. ‘In recent years, the people in China value animal welfare more, for example, during slaughter, they (try to) use the knife to bleed quickly and they use stunning first to lower stress before slaughter’ <CH>. ‘You can see some dogs or cats are abused by people (online); someone will put the videos up, and people involved will be cursed by the public’ <CH>. Likewise, the risk of losing international export clientele was also raised; ‘(improving animal welfare offers) benefits to the business owner in terms of if they supply products for export, the customer is concerned about animal welfare, and, at same time, if they provide good animal welfare our products will be good quality’ <TL>.

Other than the benefit of risk mitigation, maintenance of current markets (domestic and international) and reducing costs through treatments and stock loss, 5/17 of the groups suggested that improving animal welfare standards would open up new markets, particularly those in export ‘in the time of globalisation and industrialisation, improved animal welfare gives better opportunity to export products’ <VN>.

Finally, in regard to financial benefits, one leader briefly touched on the possibility of increased product promotion ability in China, and another on the procedural benefit of handling calmer, less stressed animals in Thailand.

### 4.4. Human Benefits: Physical Health

In most countries, the benefits were almost entirely business- and financially focussed, except in India and to some extent Bangladesh, where more emphasis was given to protecting human health, feeding the community and community livelihood. This tied into the perceived benefit of animal welfare in the form of food safety and biosecurity. ‘(Regarding) food animals, I think if you can ensure animal welfare, the food will be safe’ <BA>. 

The ties between animal welfare and human welfare, in the shape of the One Welfare initiative, was well perceived in India, with prevalent comments such as ‘animals and human welfare is the same’ <IN>. ‘Indian people live so close to animals so there’s a lot of mixing…not like in Western countries where animals are not living in close proximity…even when we go to work we meet so many animals on the road including dogs, cats, buffaloes, so there is a lot of interaction between humans and animals. Because of that, there is a lot of linking between human and AW’ <IN>. ‘If animals are healthy and happy, humans will benefit…promoting animal welfare means humans also gain welfare’ <IN>. ‘Human and animal welfare is tied…. (for example), rabies is transferred from animal to human…but still street dogs are not vaccinated’ <IN>. ‘One health; the health of animals and health of humans are interlinked… we need to improve animal health to improve human health as many diseases are zoonotic’ <IN>. ‘Also, saving money from (human) diseases…improving (the) health of animals reduces the cost of treating ourselves’ <IN>.

### 4.5. Human-Based Benefits: Psychological Wellbeing

In relation to benefits for human health, the benefits were not restricted to physical health benefits but also included benefits for human mental health. Unlike the emphasis on physical health, the inclusion of mental health benefits was not restricted to India (where it took the form of satisfying religious duty to the animals), rather, some version of mental health benefits appeared in every country. The first seemed to be the satisfaction of empathy and vindication from a perceived guilt for involvement in killing animals for both the consumers and the livestock workers themselves. ‘I am involved in animal production and I think in terms of the consumer, that if the production section is managed with good welfare then the consumer will be happy and feel good that product has come from good management, the customer will feel good about the product; hence, we can eat animals don’t feel bad’ <TL>. ‘When we do the production line and then kill the animals you feel bad about that, but at the same time that kind of animal is food for people; so we should be kind to the animals to take care of them well before they become our food’ <TL>. ‘If you look after them well, the benefit you get is good animals with less disease, increased efficiency and productivity…also, workers, livestock men, feel good doing that’ <TL>. ‘I have a small farm with free-range chickens…what I see is that the taste is very good… I enjoy watching the chickens get to live as chickens, run around flapping wings and fighting’ <MAL>. ‘I work in a layer company…most direct benefit for me personally is good feeling when I see my layers well taken care of’ <CH>.

The link between poor treatment of animals and poor treatment of other people was also recognised in this context; ‘when you provide humane treatment to animals, there is a relationship with providing humane treatment to humans’ <VN>.

### 4.6. Human-Based Benefits: Community Livelihood

How the health and wellbeing of the animals directly affects community livelihood in India and Bangladesh were benefits that were emphasised frequently in those countries, although not in any others. ‘Through history, the animal has assisted human population for livelihood development’ <IN>’. ‘In our country, we use animals for working, if welfare is ok, this will help us use animal more for ploughing’ <BA>. ‘Unlike in other countries, you can’t see animals as a separate entity, animals form an important and integral part of livelihood…If you want the benefits of improving the welfare, the prime thing is if welfare of animal is improved automatically human beings are improved’ <IN>. ‘All human beings only survive because of their livelihood, they’re dependent on this animal…if the welfare is ensured that will ensure the people’s livelihood’ <IN>. The beliefs of Hinduism were also tied to community livelihood in some instances. ‘Indians are a people who worship animals…so taking care of their welfare is equal to taking care of God, but most people are not aware of that…so you can direct things into that angle’ <IN>. ‘You cannot see the issue as two sides—cannot see animal and man as separate… consider gods of Hindus, every god is related to some animal, and actually we have a great culture of worshipping these animals; welfare is taking care of needs, people will see that as caring for gods’ <IN>.

### 4.7. Societally Based Benefits

To a lesser extent, benefits that appear to be societally focussed, bigger picture and holistic were also presented by the leaders. In some instances, particularly in China and Vietnam under the local concept of ‘ecological agriculture’, animal welfare fitted into the objectives of protecting the environment in general. This may potentially suggest that animals are seen as a part of, rather than separate from, the natural environment, from which humans are still separate. ‘if we ensure their rights, they are part of the environment, so if we improve their lifestyle and provide basic needs, they will help sustain our environment’ <VN>. 

The importance of keeping up with international progress was also raised, with improving international standing as the perceived benefit to improving animal welfare. This was specifically pertinent to government representatives in the groups. ‘From my department’s point of view, we need to achieve international recognition… without this, we will be left behind… this was evident when we had an issue regarding slaughter of animals through ESCAS (Exporter Supply Chain Assurance System) and we had to develop a whole protocol’ <ML>. Lastly, 5/17 of the groups raised the benefit of improving the human/animal relationship in general. ‘There are no definite rules and regulations in our country, no compliance with international standard…without any know-how, some people relate animal welfare to their affection, to love animals’ <BA>.

### 4.8. Animal-Based Benefits

Leaders spent the least amount of time discussing improving animal welfare for the benefit of the animals themselves; however, this was more discussed in India. Where it was discussed, it was in the context of a desire to avoid cruelty and reduce suffering. ‘We want to improve welfare for the sake of the animals’ <IN>. Other than the context of animal welfare for the animals, the other context was that of animal rights. ’Every living being has its basic rights to ensure his life is lived in proper way, and we have to ensure all basic needs and reduce the physical and psychological suffering’ <BA>.

## 5. Discussion

According to this study, the most important perceived benefit for improving animal welfare amongst livestock stakeholders is financial, primarily through increased productivity and yield from the animals in question, and improved quality of the end product (including taste in China, and not elsewhere). Other business benefits that directly or indirectly impact on the profit were ranked with high importance, including meeting customer demands and expectations, particularly with export customers, creating new markets through offering higher welfare products (again, particularly for export markets) and reducing expenses, such as treatment for disease and injury, and stock losses. Throughout the study, leaders presented benefits as if they ‘could’ be important benefits for them. So, although they were raised as important, the benefits were not necessarily without skepticism that they were necessarily achievable. Doubt around the actual existence of the perceived benefits was entirely in regard to one benefit category, increased profit, and it was particularly present in China. This suggests a need to conduct economic evaluations of financial gains that may be possible with improving individual aspects of animal welfare, ensuring reliable information is available to leaders within the livestock industry. Some well-cited studies outlined the relationship between animal welfare and economic productivity and found the attitudes of the public are intrinsically tied to this relationship [25,26] and necessarily offset by cost [12]. 

Similar work has been conducted in the field of environmental conservation and protection, with studies highlighting that financial benefits do exist and must be isolated and understood [27,28,29].

Along with environmental protection, animal protection has been hypothesized to exist in a ‘nature trifecta’ of importance to the general public [30], making it a social issue that is highly valued across borders. Examples of attitudes to animals and their perceived welfare impacting profits can be seen in select case studies. One example of this, specifically on mitigating losses rather than increasing profit, is seen with the live export industry in Australia. Media exposés highlighting animals in conditions that were distasteful to the public resulted in lobbying and temporary shut-down of the Australian live export industry in its entirety, equating to reported agricultural losses in excess of the millions [31]. On the same animal welfare issue, economic modelling found that more profit could be accessed by processing the animals in Australia, rather than sending them overseas, a solution to animal welfare concerns and an opportunity for eventual increased profit [32]. In another study, the transition from battery cages for layers hens to alternatives that increase the welfare of the birds were assessed to be economically favorable in conditions that need to be carefully measured and implemented [33]. In another case, economic modelling of the relationship between milk production and dairy cow welfare found that a herd of 100 head could increase profit margins by £10,000 (over $13,000 at the time of publishing) by implementing attainable welfare-related target rates, which is likely to have increased at the present day [34]. Likewise, profit was again related to the welfare of the dairy heifer in financial models that measured the cost of production diseases [35]. Despite the literature that argues that financial benefits exist in addressing animal welfare, making changes requires financial outlay. It is also important to note here that this paper is not affirming that financial benefits are present in all animal welfare improvement, but that, in line with the data collected, where economic modelling can be completed and financial benefit demonstrated, it could provide a largely compelling benefit that is likely to result in increased motivation to address the animal welfare change modelled. 

While financial benefits were raised as important in all countries, it was particularly the case in China and South East Asia (Malaysia, Vietnam and Thailand). All of these countries, except Malaysia, have large agriculture industries that are exporting internationally and seek to increase their export markets. However, the focus of benefits changes when looking at India and, to a lesser extent, Bangladesh, where human-focussed benefits are prioritised. Benefits such as improved human physical health through reduction of zoonotic disease risks and the relationship between animal welfare and the livelihood of the community may be the result of a culture that lives in close proximity to farm animals. As a direct result of their reverence as holy animals and their legislative protection from slaughter, cows join the ranks of commonly straying animals alongside cats and dogs in India, and interaction is frequent [36]. 

Likewise, in Bangladesh, a majority of farming is by subsistence farming, where it is commonplace that animals may be sharing a domestic environment and sometimes a home with their carers [37]. Cognitive dissonance theory refers to the pressure felt to convince oneself that immoral activities are in fact moral, to avoid uncomfortable inconsistency between attitudes and behavior [38,39]. This often unconscious human practice frequently relies on avoidance or disconnect of information or situations that result in this feeling of uncomfortable inconsistency [39]. Therefore, in the situations where avoidance or disconnection from farm-based animals is less possible, given the proximity between people and animals, cognitive dissonance from any suffering the animal may be presented with may become more difficult, and vicarious suffering may be increased. This may further explain the higher associated importance of the human–animal relationship in Bangladesh and India and the importance of benefits to the animals themselves in India.

This is also consistent with an increased concern for the ‘psychological’ wellbeing of humans and the perceived benefit that improving animal welfare will ‘make humans feel better’ in these countries, as shown by statements in this region such as ‘happy animals happy people’, and ‘if animals are healthy and happy, humans will benefit, promoting animal welfare means humans also have better welfare’. In Bangladesh, a country where cattle and buffalo are still used for work by small-farm holders, the link between the health and strength of the animal, underpinned by their welfare, and the livelihood of the community is clear. According to a comprehensive economic data analysis of 189 countries conducted by the Human Development Program at the United Nations in 2018, Malaysia (ranking 57th), Thailand (ranking 83rd) and China (ranking 86th) were considered high to very high in development, Vietnam (ranking 116) was medium, and India (ranked 130th) and Bangladesh (ranked 136th) were in the bottom of the medium development category [40]. India and Bangladesh are considered in earlier stages of development. This is consistent with the findings of this study, in which countries placed in earlier stages of development have presented the importance of human-based benefits. In this case, presenting initiatives centered on a positively impact for both human welfare and prospects for the wider community may be more likely to succeed. This is in contrast with the financially focussed profit-driven countries investigated, which are placed higher in the human development scale.

In general, benefits that are received by the animals themselves were not often presented or ranked with any great importance in most countries, despite animals being the most logical beneficiary of their welfare improvement. This could be that livestock leaders considered these benefits to be too obvious to raise; however, considering they were, on occasion, indicated along with other benefit categories, it is more likely that animal-focussed benefits were just not considered that important. Improving animal welfare for the sake of the animals is rarely a compelling argument to livestock industry leaders. One exception to this existed, i.e., again, India and Bangladesh. Improving animal welfare for the purpose of reducing suffering, respecting the animal’s rights and fulfilling the duty to appropriately consider the care of other living species could be partially attributable to the pervasion of Hindu religious beliefs in India and again due to physical proximity to the animals resulting in more developed empathy in Bangladesh. The notion of Ahimsa, non-violence, in Hinduism includes all life, and appropriate treatment of animals is tied to the tenet of Karma, where causing ill to another will result in ill to oneself. Inherently tied to rebirth, Hindus believe they may be reborn as an animal, and an animal may be reborn as a human, the specifications of which depend on their state of karma [41]. Lastly, the Vedas describe the code of sarva–bhuta–hita (devotion to the good of all creatures) [42]. 

Surprisingly, benefits to addressing animal welfare with the purpose of managing branded images and avoiding negative media and even market collapse, as has been seen with the livestock export industry in Australia [15,43,44], did not appear to be mentioned with any significance in this study. This may be due to a reduced concern in Asian countries that citizens may lobby and protest in an attempt to challenge an industry. This is potentially underpinned by the cultural dimension of ‘power distance’ (the degree to which a hierarchy and the directions provided by it are accepted without question), which is often higher in Asian countries [45]. This may also be attributable to a greater concern for maintaining economic stability as compared to western countries that enjoy a higher development ranking.

Previous research has demonstrated the importance of valuating potential changes to practices on animal welfare grounds by estimating and understanding the benefits and the costs in doing so [12,26]. Understanding the value citizens place on animal welfare benefits is deemed worth of exploration, as it directly relates to the indirect loss of profit for the industry [25]. Likewise, a better understanding of the strength of animal welfare benefits according to the livestock industry itself could have great utility. 

The findings of this study suggest potential grounds for presenting more compelling mutual benefits to livestock industry leaders when seeking to improve farm animal welfare internationally. By applying this information and creating education and awareness initiatives in line with benefits that are more likely to appeal to the livestock community, it is likely that an increased engagement with animal welfare initiatives will be seen. For countries within this study, that includes the creation and presentation of reliable data sets that demonstrate profit opportunities where they exist, and an increased effort to reach business owners and senior managers in production companies with this information.

## 6. Conclusions

This study explored benefits of improving animal welfare as perceived by livestock stakeholders in China, Vietnam, Thailand, Malaysia, India and Bangladesh. Although the overarching importance of benefits that yield financial gain was shared across all countries, mostly through improved productivity of the animals and improved product quality, regional differences were present. This was most noticeably the case with India and Bangladesh being more concerned with human- and community-focussed benefits of improving animal welfare and the tie between human and animal welfare by reducing zoonotic risks, but also in regard to the potential increase in human psychological welfare by observing the animals in more positive states. Animal-focussed benefits were not presented with any significance by the livestock leaders included in this study, with the exception of India and Bangladesh, suggesting that improving animal welfare for the sake of the animals is unlikely to be a compelling reason to act in most cases of livestock enterprise, unless it is directly related to the productive output of the animal or another financial indicator such as reducing the risk of stock losses. This study does not investigate the presence of any benefits of improving animal welfare, rather, it investigates which benefits, should they be present, would be most valued by stakeholders. If applied to international animal welfare initiatives with the purpose of finding mutual benefits to initiate collaborations, this founding information could be useful. In addition, if the more compelling benefits presented in this study can be investigated and demonstrated, it is suggested that stakeholders will be more likely to engage in change to improve animal welfare. 

## Figures and Tables

**Table 1:** Location of focus groups and abbreviation codes used in quote citations.

Country	Abbreviated Code	City/Town	Participant N	
**China**	CH	Guangzhou	7	23
		Zhengzhou	7	
		Beijing	9	
**Vietnam**	VN	Hanoi	7	20
		Ban Me Thout	5	
		Ho Chi Minh City	8	
**Thailand**	TL	Bangkok	10	19
		Khon Kaen	3	
		Chiang Mai	6	
**Malaysia**	MAL	Negeri Sembilan	6	19
		Kuala Lumpur Selangor	13	
**India**	IN	Banglaore	6	15
		Kolkata	5	
		Trivandrum	4	
**Bangladesh**	BA	Dhaka	13	43
		Savar	13	
		Mymensingh	17	

**Table 2:** Breakdown of stakeholder participant roles within the livestock industry, by country.

Country	Stakeholder Role			
	Private Industry Leaders	Private Industry Veterinarians	Government Representatives	Agricultural Academics
**China**	15	0	1	9
**Vietnam**	4	3	13	1
**Thailand**	11	4	2	2
**Malaysia**	9	5	5	1
**India**	3	5	1	6
**Bangladesh**	4	2	17	21

**Table 3:** Benefits identified by participants in each region, in each country, presented in order from the most frequently identified benefit to the least cited (top to bottom).

	China			Vietnam			Thailand			Malaysia		India			Bangladesh		
	Beijing	Guangzhou	Zhengzhou	Hanoi	Ho Chi Minh City	Ban Me Thout	Bang-kok	Chiang Mai	Khon Kaen	Kuala Lumpur	Negeri Sembil-an	Kolkata	Bangal-ore	Trivan-drum	Dhaka	Myme-nsingh	Savar
Productivity of animals	X		X	X	X	X		X		X	X	X	X		X	X	X
Improve quality of meat or animal product	X	X	X	X	X	X	X	X	X	X			X	X			X
Reduce disease and injury and treatment costs		X		X	X		X	X			X	X		X		X	X
Increased revenue/profit		X			X				X		X		X	X	X	X	
Avoid cruelty and reduce animal suffering	X		X			X		X		X		X	X			X	X
Human health/zoonosis					X	X				X		X		X		X	
Protection of natural resources/ecosystem development	X		X			X				X		X			X		
Food safety/biosecurity						X		X		X				X	X	X	
International trade opportunities	X	X			X				X								X
Stronger/healthier animals	X		X						X							X	
People feel better for the animals		X	X		X			X						X			
Improve human/animal relationship				X	X							X		X			X
Addressing the animals’ rights/sanctity of life												X	X		X		
Improved community livelihood												X	X	X			
Public concern/consumer confidence	X		X				X										
Relationship between way humans and animals are treated, tie to human welfare				X		X											
Improved taste of animal product		X	X								X						
International recognition (not being left behind)			X								X						
Allowing natural behaviour of animals											X						X
Compliant with international regulation					X												X
Human responsibility to give a good life								X						X	X		
Lower mortality					X			X									
Ease of handling calmer animals							X										
Improved commercial promotion	X																

**Note:** ‘X’ signifies the presence of the theme in the focus group session in that region. 
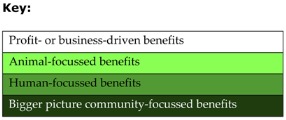

**Table 4:** Ranking of importance of benefits, by country.

Rank	China	Vietnam	Thailand	Malaysia	India	Bangladesh
**1**	Improve quality of meat or animal product	Improve quality of meat or animal product	Improve quality of meat or animal product	Productivity of animals	Avoid cruelty and reduce animal suffering	Productivity of animals
**2**	Stronger/healthier animals	Productivity of animals	Reduce disease and injury and treatment costs	Increased revenue/profit	Improved community livelihood	Reduce disease and injury and treatment costs
**3**	Protection of natural resources/sustainable development	Reduce disease and injury and treatment costs	Stronger/healthier animals	Food safety/biosecurity	Human health/zoonosis	Food safety/biosecurity
**4**	Productivity of animals	Human health/zoonosis	Human responsibility to give a good life	Improve quality of meat or animal product	Reduce disease and injury and treatment costs	Increased revenue/profit
**5**	People feel better for the animals	Improve human/animal relationship	Increased revenue/profit	Reduce disease and injury and treatment costs	Addressing the animals’ rights/sanctity of life	Stronger/healthier animals
**6**	Public concern/consumer confidence	Relationship between way humans and animals are treated, tie to human welfare	Avoid cruelty and reduce animal suffering	Human health/zoonosis	Productivity of animals	Avoid cruelty and reduce animal suffering
**7**	Increased revenue/profit	Protection of natural resources/sustainable development	Food safety/biosecurity	Avoid cruelty and reduce animal suffering	People feel better for the animals	Addressing the animals’ rights/sanctity of life
**8**	Improved taste of animal product	Food safety/biosecurity	Ease of handling calmer animals	Improved taste of animal product	Increased revenue/profit	Improve quality of meat or animal product
**9**	Avoid cruelty and reduce animal suffering	Increased revenue/profit	International trade opportunities	Protection of natural resources/sustainable development	Food safety/biosecurity	Protection of natural resources/ecosystem development
**10**	Reduce disease and injury and treatment costs	Avoid cruelty and reduce animal suffering	People feel better for the animals	International recognition (not being left behind)	Improve human/animal relationship	Allowing natural behaviour of animals
**11**	International trade opportunities	International trade opportunities	Public concern/consumer confidence	Allowing natural behaviour of animals	Human responsibility to give a good life	Compliant with international regulation
**12**	Improved commercial promotion	People feel better for the animals	Productivity of animals		Improve quality of meat or animal product	Human health/zoonosis
**13**	International recognition (not being left behind)	Compliant with international regulation	Lower mortality		Protection of natural resources/ecosystem development	Improve human/animal relationship
**14**		Lower mortality				International trade opportunities

Notes: Shaded cells indicate that the benefits were ranked equally.
